# Heart Rate Changes in Response to Mechanical Pressure Stimulation of Skeletal Muscles Are Mediated by Cardiac Sympathetic Nerve Activity

**DOI:** 10.3389/fnins.2016.00614

**Published:** 2017-01-10

**Authors:** Nobuhiro Watanabe, Harumi Hotta

**Affiliations:** Department of Autonomic Neuroscience, Tokyo Metropolitan Institute of GerontologyTokyo, Japan

**Keywords:** skeletal muscles, mechanical pressure stimulation, somatocardiovascular reflexes, heart rate, cardiac sympathetic nerve, rats

## Abstract

Stimulation of mechanoreceptors in skeletal muscles such as contraction and stretch elicits reflexive autonomic nervous system changes which impact cardiovascular control. There are pressure-sensitive mechanoreceptors in skeletal muscles. Mechanical pressure stimulation of skeletal muscles can induce reflex changes in heart rate (HR) and blood pressure, although the neural mechanisms underlying this effect are unclear. We examined the contribution of cardiac autonomic nerves to HR responses induced by mechanical pressure stimulation (30 s, ~10 N/cm^2^) of calf muscles in isoflurane-anesthetized rats. Animals were artificially ventilated and kept warm using a heating pad and lamp, and respiration and core body temperature were maintained within physiological ranges. Mechanical stimulation was applied using a stimulation probe 6 mm in diameter with a flat surface. Cardiac sympathetic and vagus nerves were blocked to test the contribution of the autonomic nerves. For sympathetic nerve block, bilateral stellate ganglia, and cervical sympathetic nerves were surgically sectioned, and for vagus nerve block, the nerve was bilaterally severed. In addition, mass discharges of cardiac sympathetic efferent nerve were electrophysiologically recorded. Mechanical stimulation increased or decreased HR in autonomic nerve-intact rats (range: −56 to +10 bpm), and the responses were negatively correlated with pre-stimulus HR (*r* = −0.65, *p* = 0.001). Stimulation-induced HR responses were markedly attenuated by blocking the cardiac sympathetic nerve (range: −9 to +3 bpm, *p* < 0.0001) but not the vagus nerve (range: −75 to +30 bpm, *p* = 0.17). In the experiments with cardiac sympathetic efferent nerve activity recordings, mechanical stimulation increased, or decreased the frequency of sympathetic nerve activity in parallel with HR (*r* = 0.77, *p* = 0.0004). Furthermore, the changes in sympathetic nerve activity were negatively correlated with its tonic level (*r* = −0.62, *p* = 0.0066). These results suggest that cardiac sympathetic nerve activity regulates HR responses to muscle mechanical pressure stimulation and the direction of HR responses depends on the tonic level of the nerve activity, i.e., bradycardia occurs when the tonic activity is high and tachycardia occurs when the activity is low.

## Introduction

Somatosensory stimulation reflexively elicits autonomic nervous activity changes and affects cardiovascular control (somatocardiovascular reflexes) in anesthetized animals, in whom consciousness and emotions that are potentially influential are eliminated by the administration of anesthesia (Sato et al., 1997; Watanabe et al., 2015). One characteristic of somatocardiovascular reflexes is that evoked responses differ depending on the type of stimulation. For example, a strong pinch of the skin (Kimura et al., 1995; Sato et al., 1997; Suzuki et al., 2004) and noxious thermal stimulation (Kaufman et al., 1977) generally induce tachycardiac and pressor responses. In contrast, brushing and non-noxious thermal stimulation do not lead to heart rate (HR) responses or provide only a small response (Kaufman et al., 1977; Sato et al., 1997). In addition to the skin stimulation mentioned above, some skeletal muscle stimulations evoke cardiovascular responses (Sato et al., 1981, 1982; Kannan et al., 1988; Stebbins et al., 1988; Sato et al., 1997). For example, static muscle contraction and stretch induce tachycardiac and pressor responses (Coote et al., 1971; Kannan et al., 1988; Stebbins et al., 1988), whereas vibratory stimulation does not influence HR and blood pressure (Kannan et al., 1988; Sato et al., 1997). These cardiovascular responses to skin and muscle stimulation are mainly attributed to the excitation of group III and IV afferent fibers (Sato et al., 1997). HR and blood pressure responses to contraction and stretching of skeletal muscles are considered important cardiovascular regulatory mechanisms during exercise (Coote et al., 1971; Murphy et al., 2011). As the efferent of neural mechanisms, the cardiac, renal, and adrenal sympathetic nerve activities are enhanced by the static contraction of the hindlimb muscles (Matsukawa et al., 1990, 1994; Vissing et al., 1991; Koba et al., 2008).

Some mechanoreceptors in skeletal muscles are distinguished from muscle contraction- or stretch-sensitive units based on high sensitivity to pressure stimulation (Paintal, 1960; Mense and Meyer, 1985). Hence, it is possible that cardiovascular responses elicited by pressure stimulation differ from those elicited by muscle contraction. Stebbins et al. (1988) reported that static contraction of calf muscles in anesthetized cats increased HR and blood pressure (by 10 bpm and 20 mmHg, respectively), whereas the constant pressure stimulation applied externally did not change the HR and only marginally increased the blood pressure (5–10 mmHg). Uchida et al. (2003) reported that static pressure to calf muscles (~5–8 N/cm^2^) induced a depressor response in anesthetized rats. In a study by Tallarida et al. (1981), “squeeze” stimulation applied to the calf muscles caused tachycardiac and pressor responses in anesthetized rabbits, although the precise intensity of stimulation was not determined. Despite studies reporting cardiovascular responses to mechanical pressure stimulation of skeletal muscles, the neural mechanisms underlying this effect are undetermined to date.

Sustained muscle contraction generally induces a pressor response, whereas mechanical pressure stimulation of calf muscles can induce both pressor (Tallarida et al., 1981; Stebbins et al., 1988) and depressor (Uchida et al., 2003) responses. Even under controlled experimental conditions, it was reported that electrical stimulation of muscle afferents (Sato et al., 1981), bradykinin infusion to hindlimb muscles (Sato et al., 1982), and acupuncture-like stimulation to hindlimb muscles (Ohsawa et al., 1995) could induce tachycardiac and bradycardiac or pressor and depressor responses. The reasons for these bidirectional cardiovascular responses have not been studied. It has been documented that deeper anesthesia is more likely to induce bradycardiac and depressor responses (Gibbs et al., 1989; Sato et al., 1997). The depth of anesthesia generally affects the resting levels of HR and blood pressure.

Therefore, there were two aims of the present study. The first aim was to elucidate the contribution of cardiac autonomic nerves to HR responses induced by mechanical pressure stimulation of skeletal muscles. The second aim was to examine whether the resting (pre-stimulus) level of HR influences the direction of HR responses to skeletal muscle mechanical pressure stimulation. To maintain the constant level of anesthesia through data recordings, we used inhalation anesthesia (isoflurane) in the present study.

## Materials and methods

### Animals

Experiments in the present study were performed on Wistar male rats (4–7 months, *n* = 18) bred at the Tokyo Metropolitan Institute of Gerontology. All study protocols were approved by the animal care and use committee of the Tokyo Metropolitan Institute of Gerontology and conformed to the Guiding Principles for the Care and Use of Animals in the Field of Physiological Sciences.

Rats were anesthetized using isoflurane (Escain, Mylan Inc., Canonsburg, PA, USA). Isoflurane was vaporized by gas (O_2_ 30%, N_2_ 70%) using a vaporizer (Sigma Delta, Penlon Ltd., Abingdon, UK). The inspiratory concentration of isoflurane was set at 4% for anesthesia induction and maintained at 2.5–3.0% during surgery. Throughout data recording, isoflurane was maintained at 1.2–1.4%, which is sufficient to eliminate the corneal reflex. In all rats, catheters were implanted into the common carotid artery to continuously record arterial pressure and into the jugular vein to administer drugs and supplemental fluids. The trachea was cannulated and rats were artificially ventilated (SN-480-7; Shinano Seisakusho, Tokyo, Japan). Respiration was controlled to maintain end-tidal CO_2_ at ~3.0% (Capnostream™ 20P, Covidien, Minneapolis, MN, USA). Rectal temperature was maintained at 37.0–37.5°C using an automatically regulated heating pad and lamp (ATB-1100; Nihon Kohden, Tokyo, Japan).

HR was calculated based on recorded arterial pressure waveforms with a time constant of 5 s (Spike 2; Cambridge Electronic Design, Cambridge, England). Mean arterial pressure was obtained by smoothing arterial pressure waveforms with a time constant of 5 s (Spike 2). HR and blood pressure were continuously monitored during experiment, and mechanical stimulation (see below) was applied after confirming that HR and blood pressure were stable for at least 1 min. The HR response (ΔHR) to stimulation was determined to be the maximum within 1 min after termination of mechanical stimulation relative to the average HR over 1 min before stimulation. The presence of a response was defined as the ΔHR value exceeding by twofold the spontaneous variability during 1 min of pre-stimulus recording (i.e., mean value ± twice the standard deviation).

### Mechanical stimulation of muscle and skin

Mechanical pressure stimulation was applied to the calf muscles according to previous reports (Graven-Nielsen et al., 2004; Takahashi et al., 2005; Mizumura and Taguchi, 2016). A stimulation probe 6 mm in diameter with a flat surface (contact area, ~28 mm^2^) was applied perpendicularly to the skin over the center of the inner calf with a weight of ~290 g (≈10 N/cm^2^) for 30 s. The stimulation intensity was based on previous studies showing that this pressure level increases the single unit activities of group III and IV calf muscle afferents (Berberich et al., 1988; Hoheisel et al., 2005). In a pilot study, we confirmed that mechanical pressure at 10 N/cm^2^ induced clearer HR changes than stimulation at lower intensities (2–5 N/cm^2^). The fur at the site of pressure stimulation was trimmed using a conventional clipper. Noxious mechanical stimulation was applied to the skin by pinching the hindpaw for 30 s using a surgical clamp (~3 kg; Araki et al., 1984).

### Autonomic nerve block

To identify the nerve pathway that contributes to the HR response evoked by calf muscle pressure stimulation, the influence of selective autonomic nerve block was examined. To block sympathetic nerves innervating the heart, bilateral stellate ganglia and cervical sympathetic nerves were surgically severed before the end of surgery in three rats. The second costal bone was sectioned before the stellate ganglion was crushed. To block vagus nerves, bilateral vagus nerves were surgically sectioned at the cervical level in 6 rats. In another rat, vagus efferents were pharmacologically blocked by intravenous administration of the blood–brain barrier impermeable muscarinic receptor blocker atropine methyl nitrate (2 mg/kg; Overton, 1993) purchased from Sigma-Aldrich (St. Louis, MO, USA). We confirmed that the dose of atropine was enough to prevent bradycardiac responses induced by electrical stimulation of vagus efferent nerve (Hotta et al., 2010b) at the end of the experiment. The vagotomy was performed before experiment in one rat. In the other rats, vagus nerves were blocked during experiment, and effects of mechanical stimuli were examined both before and after blocking of the vagus nerve.

### Cardiac sympathetic efferent nerve activity recording

Cardiac sympathetic efferent nerve activity was recorded in four rats by methods described in our previous study (Hotta et al., 2010a). In brief, anesthetized rats were placed in the supine position and the right second costal bone was sectioned. The right inferior cardiac sympathetic nerve was exposed, sectioned as close to the heart as possible, and isolated from surrounding connective tissue. The dissected nerve was covered with paraffin oil. The central cut end of the nerve was placed on platinum–iridium bipolar hook electrodes and mass discharges were recorded. To prevent noise contamination due to muscle contraction, rats were immobilized by intravenous administration of gallamine triethiodide (20 mg/kg). For CSNA recording, the vagus nerves were sectioned to prevent contamination of vagus nerve activity.

Nerve activity was amplified 1000 × (MEG-6100, Nihon Kohden, Tokyo, Japan), filtered (bandpass filter: 150 Hz–3 kHz), and monitored visually on an oscilloscope and auditorily through loudspeakers. The amplified signals were digitized at 20 kHz (Micro 1401 mkII; Cambridge Electronic Design) and stored on a personal computer for offline analyses. Spikes of the nerve were discriminated from background noise based on the amplitude of signals, and the number of the spikes was counted every 5 s (Spike 2).

### Statistical analyses

HR and mean arterial pressure values obtained before and after the onset of stimulation were compared by paired *t*-test or Wilcoxon matched-pairs signed rank test depending on the normality of the data distribution (Prism 6; GraphPad Software Inc., La Jolla, CA, USA). Difference in variance of pre-stimulus HR and ΔHR under autonomic block conditions were examined by the *F*-test. Correlation strength was analyzed by calculating Spearman's coefficient. Statistical significance was set at *p* < 0.05. Data are expressed as mean ± standard deviation unless otherwise stated.

## Results

### HR and blood pressure responses to mechanical pressure stimulation of the calf muscles

Resting HR of anesthetized rats (*n* = 9) prior to applying mechanical stimulation ranged from 259 to 459 bpm. Resting HR is relatively stable, but exhibited periodic step-like changes to higher or lower levels of HR (Yli-Hankala and Jäntti, 1990). In these 9 rats, 22 trials of mechanical pressure stimulation were applied to the calf. HR increased in 9 trials and decreased in 12 trials, with no change in one trial. Overall, HR significantly decreased in response to calf pressure stimulation (from 356.3 ± 59.1 to 346.8 ± 48.9 bpm, *p* = 0.049 by paired *t*-test, range −56 and +10 bpm).

**Figure 1:**
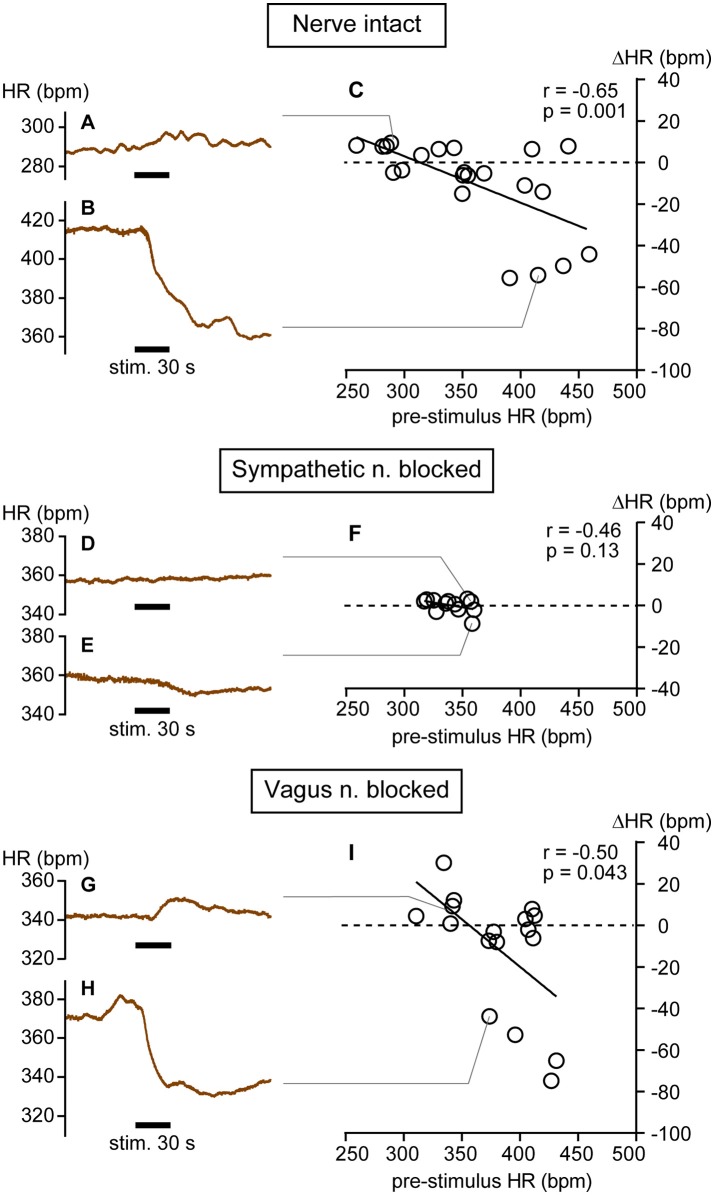
**Heart rate (HR) changes in response to calf muscle pressure stimulation in nerve intact (A–C), sympathetic nerve blocked (D–F), and vagus nerve blocked (G–I) conditions. (A,B)** Bidirectional changes in heart rate (HR) in response to calf pressure stimulation in anesthetized rats with intact autonomic innervation of the heart (Nerve intact). **(C)** Correlation between pre-stimulus HR and HR responses (ΔHR). Each individual maximal ΔHR is plotted as an open circle. **(D–F)** Data obtained in rats with sympathetic nerve blocked (Sympathetic n. blocked). **(G–I)** Data obtained in rats with vagus nerve blocked (Vagus n. blocked). *r* = Spearman's correlation coefficient.

Examples of increasing and decreasing responses are shown in Figures 1A,B, respectively. In response to pressure stimulation, HR increased slightly (e.g., maximal ΔHR = 10 bpm in Figure 1A) from relatively low pre-stimulus HR (e.g., 288 bpm in Figure 1A) or decreased more substantially (e.g., maximal ΔHR = −54 bpm in Figure 1B) from a relatively high pre-stimulus HR (e.g., 415 bpm in Figure 1B). The maximal increase or decrease was attained within a minute following the cessation of stimulation. Bradycardiac responses of >40 bpm were evoked when pre-stimulus HR was >390 bpm and smaller HR responses were produced when HR was <390 bpm. There was a significant negative correlation between pre-stimulus HR and ΔHR (*r* = −0.65, *p* = 0.001; Figure 1C).

We also applied mechanical pressure stimulation directly to muscle (three trials in a rat) after carefully removing the overlying skin. Both increasing (from 342 to 349 bpm) and decreasing (from 349 to 328 bpm and from 339 to 305 bpm) responses were observed. The correlation between pre-stimulus HR and ΔHR was maintained. Thus, cutaneous afferents are not solely responsible for these HR responses.

On average, mean arterial pressure decreased in response to stimulation (from 113.8 ± 38.6 to 103.7 ± 30.9 mmHg, *p* = 0.033 by Wilcoxon matched-pairs signed rank test, 22 trials). Pre-stimulus mean arterial pressure level was negatively correlated with mean arterial pressure changes (*r* = −0.72, *p* = 0.0002). In majority of trials, HR and mean arterial pressure changed in the same direction (i.e., tachycardiac and pressor responses in Figure 1A and Supplementary Figure 1A or bradycardiac and depressor responses in Figure 1B and Supplementary Figure 1B). There was a significant positive correlation between HR and mean arterial pressure changes (*r* = 0.83, *p* < 0.0001, Supplementary Figure 1C).

### Influence of autonomic nerve block on pre-stimulus HR and response to calf pressure stimulation

In three rats, cardiac sympathetic nerves were blocked and 12 trials of mechanical pressure stimulation were conducted. Overall, HR did not change in response to mechanical stimulation (from 340.3 ± 15.5 to 340.3 ± 14.4 bpm, *p* = 0.97 by paired *t*-test). Spontaneous fluctuations of HR during the pre-stimulus period were quite small in this condition (Figures 1D,E); thus, even very small changes in HR were distinguishable (e.g., from 357 to 359 bpm in Figure 1D and from 359 to 350 bpm in Figure 1E). Under sympathetic nerve block, both the range of pre-stimulus HR (317–360 bpm) and the ΔHR (−9 to +3 bpm) were significantly smaller than those in rats with intact autonomic nerves (both *p* < 0.0001 by *F*-test). There was no significant correlation between pre-stimulus HR and HR response (*r* = −0.46, *p* = 0.13; Figure 1F), implicating sympathetic nerve in these HR responses to mechanical pressure stimulation of calf muscle.

In seven rats, the vagus nerve was blocked and 17 trials of mechanical pressure stimulation to calf muscle were performed. In this condition, bidirectional HR responses (range: −75 to +30 bpm; Figures 1G–I) were observed and the range of ΔHR was not different from that in rats with intact autonomic nerves (*p* = 0.17 by *F*-test). A significant negative correlation between pre-stimulus HR and ΔHR remained (*r* = −0.50, *p* = 0.043; Figure 1I), although the range of pre-stimulus HR (311–431 bpm) was narrower than that in autonomic nerve intact rats (*p* = 0.048 by *F*-test). Bradycardiac responses of >40 bpm were produced when pre-stimulus HR was >370 bpm. This inverse correlation between pre-stimulus HR and ΔHR was also observed in one of the seven rats in whom the vagus efferent nerve was pharmacologically blocked by atropine administration rather than transection. Results obtained from this atropine-treated rat are grouped together with those obtained from the vagus nerve-severed rats (Figure 1I). When averaged, HR did not change in response to mechanical stimulation (from 380.9 ± 36.0 to 369.7 ± 31.2 bpm, *p* = 0.14 by paired *t*-test).

### Response of cardiac efferent nerve activity to calf muscle pressure stimulation

**Figure 2:**
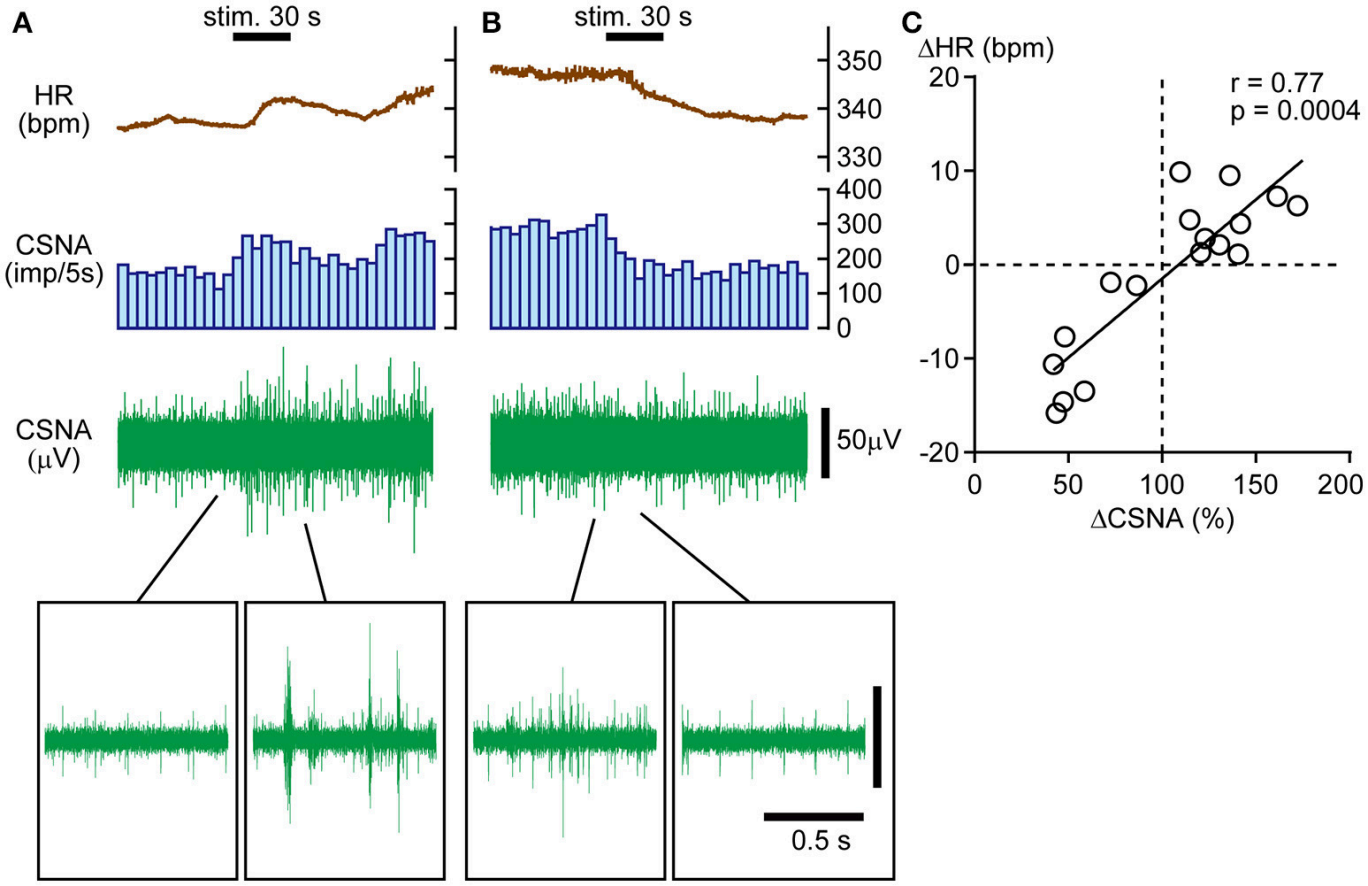
**Direct association between bidirectional heart rate (HR) and cardiac sympathetic efferent nerve activity (CSNA) changes in response to calf pressure stimulation. (A,B)** Sample records showing increased **(A)** or decreased **(B)** CSNA with parallel changes in HR. The histogram illustrates the nerve discharge rate every 5 s. Raw signals are shown in the upper to lower rows. Insets in **(A,B)** show enlarged views of nerve activity. **(C)** Positive correlation between ΔCSNA and ΔHR. *r* = Spearman's correlation coefficient.

The results presented above suggest that HR responses to calf pressure stimulation are mediated primarily by changes in CSNA. Hence, we electrophysiologically recorded CSNA and obtained nerve responses to calf muscle pressure stimulation. In four rats, CSNA was recorded during 17 trials of mechanical pressure stimulation. Similar to HR responses, CSNA responses (ΔCSNA) were bidirectional, increasing in 10 trials and decreasing in 6 trials with one no response trial. Sample CSNA recordings with simultaneous HR monitoring from the same rat are shown in Figures 2A,B. In Figure 2A, CSNA started to increase immediately after the onset of the calf pressure stimulation and peaked at 172.2% of pre-stimulation value. In Figure 2B, CSNA decreased immediately after the onset of stimulation to 41.9% below pre-stimulus value at 20 s after cessation of stimulation. In both cases, the direction of the HR change paralleled that of CSNA, and there was a strong positive correlation between ΔHR and ΔCSNA (*r* = 0.77, *p* = 0.0004; Figure 2C).

**Figure 3:**
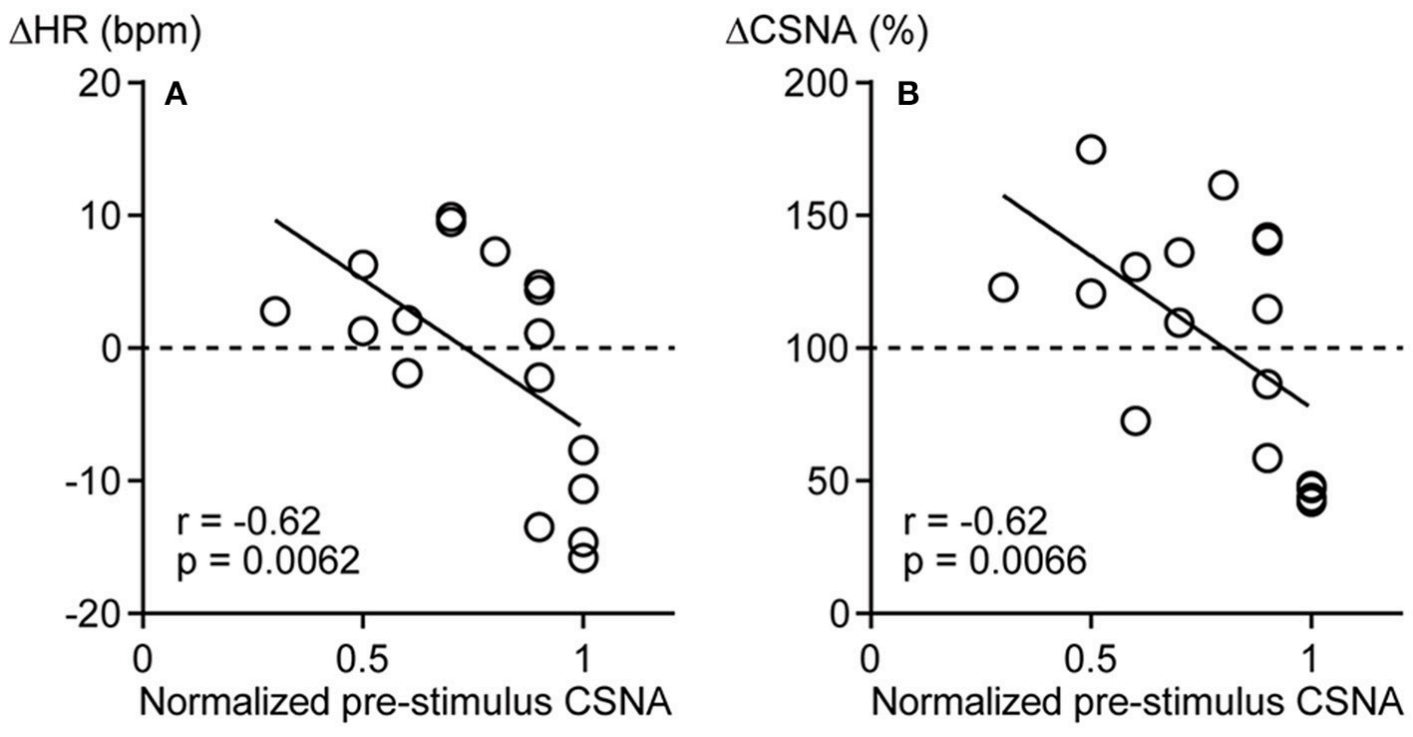
**Negative correlations between pre-stimulus cardiac sympathetic efferent nerve activity (CSNA) and changes in heart rate (ΔHR, A) and sympathetic nerve activity (ΔCSNA, B) in response to calf muscle pressure stimulation**. The highest frequency value of CSNA across all trials was used to normalize the level of CSNA within each rat and expressed as 1. *r* = Spearman's correlation coefficient.

To identify factors influencing the direction of the CSNA response to pressure stimulation, the correlations of pre-stimulus CSNA level with ΔHR and ΔCSNA were examined. To pool pre-stimulus CSNA data from different rats, CSNA was normalized to the highest frequency value across all trials within each individual (and set to 1). The normalized pre-stimulus CSNA was significantly and negatively correlated with both ΔHR and ΔCSNA responses to calf muscle pressure stimulation (*r* = −0.62, *p* = 0.0062 and *r* = −0.62, *p* = 0.0066, respectively; Figures 3A,B). Thus, mechanical pressure stimulation of skeletal muscle produces decreased HR and CSNA when tonic CSNA is high and increased HR and CSNA when tonic CSNA is relatively low.

### Correlation between pre-stimulus HR and HR response to pinch stimulation of the hindpaw

**Figure 4:**
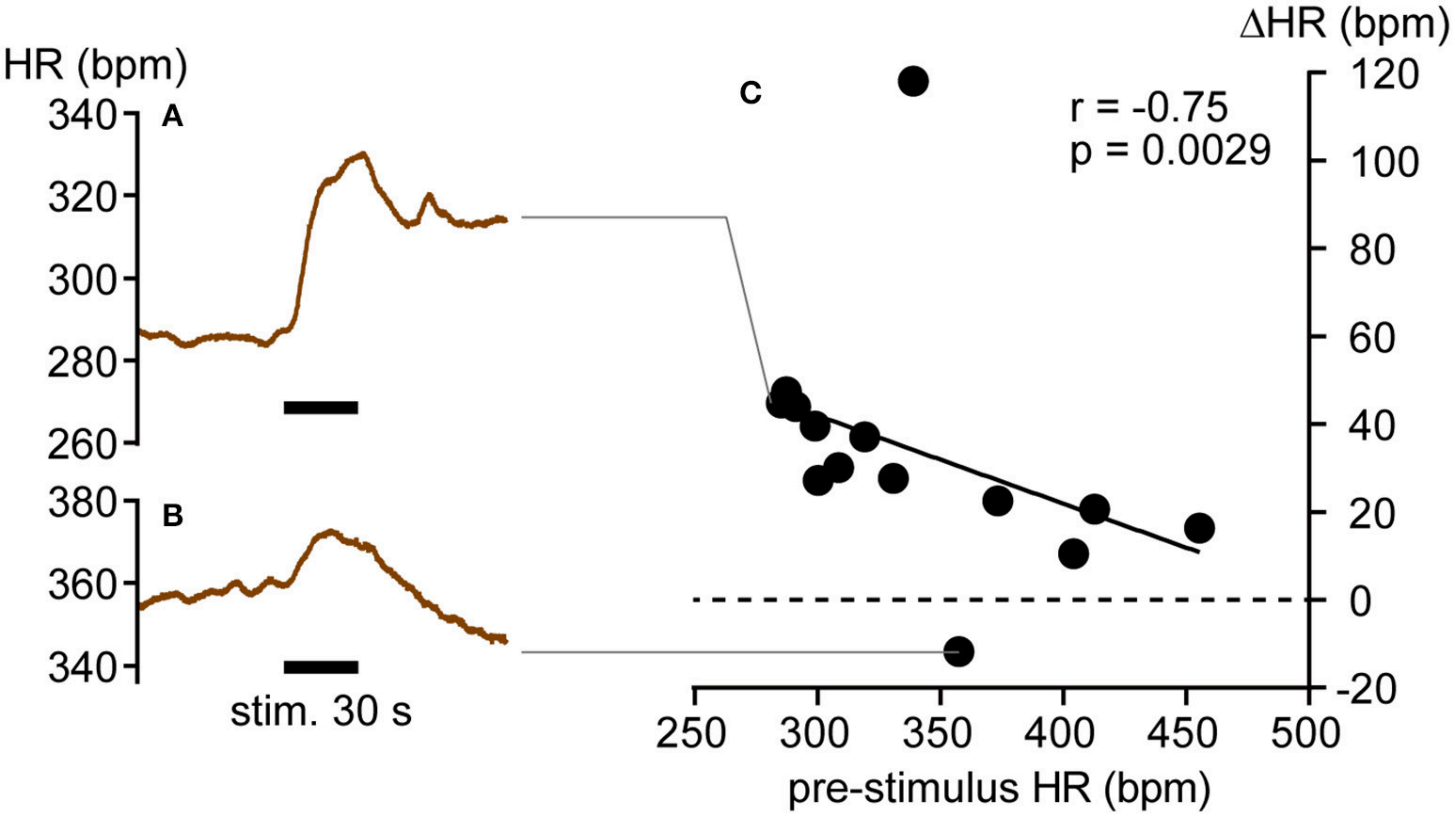
**Heart rate responses (ΔHR) to hindpaw pinch stimulation. (A,B)** HR increase **(A)** and decrease **(B)** in response to pinch stimulation. **(C)** Negative correlation between individual ΔHR values (closed circles) and pre-stimulus HR. *r* = Spearman's correlation coefficient.

We then examined whether HR responses to pinch stimulation were also influenced by the level of pre-stimulus HR. In 6 rats with intact autonomic nerves, 14 trials were performed. Overall, HR and mean arterial pressure significantly increased (from 340.3 ± 53.4 to 374.2 ± 50.5 bpm, *p* = 0.0004, and from 96.3 ± 37.9 to 125.1 ± 27.3 mmHg, *p* = 0.0052, respectively, by Wilcoxon matched-pairs signed rank test). As shown in Figure 4A, HR increased immediately after the onset of pinch stimulation and remained above the pre-stimulus level for more than 3 min after the cessation of stimulation in most trials. In 13 of 14 trials, HR increased in response to pinch stimulation (Figures 4A,C). In the other trial, HR increased immediately after the onset of pinch stimulation and exhibited a larger decrease following the termination of the stimulation (Figures 4B,C). Like muscle stimulation, there was a significant negative correlation between pre-stimulus HR and the ΔHR responses to pinch stimulation (*r* = −0.75, *p* = 0.0029; Figure 4C).

## Discussion

The present results demonstrate that (1) calf muscle pressure stimulation induces tachycardiac or bradycardiac responses by regulating CSNA because HR responses to pressure stimulation in cardiac sympathetic nerve blocked condition were marginal and the direction of the HR change paralleled that of CSNA and (2) the direction of the HR change is dependent on the tonic level of CSNA, i.e., bradycardia occurs when the tonic activity is high and tachycardia occurs when it is low.

### HR responses to mechanical pressure stimulation of the calf muscle were mediated by cardiac sympathetic nerves

The present observations that HR responses to calf muscle pressure stimulation were suppressed by cardiac sympathetic nerve block and strongly correlated with CSNA indicate that these responses are mediated predominantly by changes in CSNA. Furthermore, similar results were observed with direct muscle pressure after skin removal, implicating muscle mechanoreceptor afferent in this response and precluding a necessary contribution from cutaneous afferents. Moreover, since HR and blood pressure usually changed in the same direction, the HR responses likely originated from sensory inputs from the calf muscle rather than from a secondary response to blood pressure changes (i.e., baroreflexes).

In contrast to sympathetic nerve, the contribution of vagus nerve appeared negligible because there was no significant influence of vagus nerve transection on the HR response. Tallarida et al. (1981) reported that pressure stimulation of the calf muscles increased HR and this tachycardiac response was blocked by the catecholamine release inhibitor guanethidine in anesthetized vagotomized rabbits. Although, the influence of vagotomy was not reported in their study, presence of the tachycardiac response under vagotomy and blockade by inhibition of postganglionic sympathetic norepinephrine release are fundamentally consistent with our results. However, there remains a possibility that the vagus nerve may contribute to HR responses in the unanesthetized condition because cardiac vagus nerve is more susceptible to anesthetics than sympathetic nerve (Sato et al., 1997). In conscious humans, mechanical muscle stimulation by stretch is considered to increase HR through vagal withdrawal (Gladwell et al., 2005).

### Tonic level of cardiac sympathetic nerve activity determines the direction of the HR response

The directions of changes in HR and blood pressure induced by muscle stimulation are reported to be affected by experimental conditions such as the depth of anesthesia (Ohsawa et al., 1995; Sato et al., 1997). However, the inhaled concentration of isoflurane was kept constant during data recording in the present study, indicating bidirectional HR responses (bradycardia or tachycardia) to the mechanical pressure stimulation were not due to shifts in anesthesia level. Rather, response direction was dependent on the pre-stimulus HR, which is a function of CSNA. Under isoflurane anesthesia, the resting HR periodically shifted (“step-like changes”) during electroencephalogram (EEG) signal pattern changes, i.e., HR increases when EEG bursts occur and decreases when EEG suppression occurs (Yli-Hankala and Jäntti, 1990). Thus, the resting level of neuronal activity in the cardiovascular center may also change periodically as manifested by the resting (pre-stimulus) HR level changes observed in the present study. However, a fundamental cause of the large variability in resting HR observed in the present study has not been determined. The range of the resting HR obtained in the present study was similar to that obtained in conscious unstrained rats (Delaunois et al., 2009; Albrecht et al., 2014; Sharp et al., 2014).

Destruction of the sympathetic inputs to the heart markedly reduced pre-stimulus HR variation, although the vagus nerve block had little effect. Thus, sympathetic inputs are the primary contributor to HR variation under these conditions. A substantial influence of sympathetic nerve block on pre-stimulus HR may be related to the inhibition of noradrenaline release from the postganglionic sympathetic terminals by the cardiac vagus nerve (Vanhoutte and Levy, 1980; Manabe et al., 1991). Thus, eliminating the tonic activity of cardiac sympathetic nerve may suppress a modulatory mechanism for resting HR driven by the vagus nerve.

We assume that the HR responses to the calf pressure stimulation are a supraspinal reflex (Sato et al., 1997). The tonic activity of sympathetic nerves innervating cardiovascular organs is thought to be generated by a core network consisting of neurons in the rostral ventrolateral medulla (RVLM), caudal ventrolateral medulla (CVLM), nucleus of the solitary tract (NTS), hypothalamus, and spinal cord (Campos and McAllen, 1997; Dampney et al., 2003; Horiuchi et al., 2004; Guyenet, 2006). Although, the central projections of pressure-sensitive muscle afferents have not been determined, muscle afferent information reportedly reaches the RVLM, CVLM, NTS, and hypothalamus (Terui et al., 1987; Kannan et al., 1988; Degtyarenko and Kaufman, 2006; McCord and Kaufman, 2010). Further, electrical stimulation to muscle afferents excited CVLM neurons and subsequently inhibited RVLM neurons (Ruggeri et al., 1995). Therefore, the direction of the HR response may be determined by the balance of excitatory and inhibitory effects on RVLM neurons. For example, at a relatively high level of RVLM neuron activity and concomitantly high CSNA, further enhancement of RVLM neuron activity by muscle afferent input may be limited due to a ceiling effect. This assumption is supported by the present result that the tachycardiac response to pinch stimulation was attenuated when pre-stimulus HR was relatively high (Figure 4). Hence, inhibition on RVLM neurons via CVLM neuron excitation can be dominant, leading to decreases in CSNA and HR. Conversely, when RVLM neuron activity and CSNA are low, pressure stimulation may be more likely to increase RVLM excitation, resulting in higher CSNA and HR.

It has been reported that resting HR level may also influence the bradycardiac response to acupuncture stimulation. Although, the afferent types may differ from those activated by mechanical pressure, there may be common mechanisms. Imai and Kitakoji (2003) showed that the degree of bradycardia induced by acupuncture stimulation in healthy volunteers was greater while sitting than when in the supine position, and sitting is associated with higher resting HR. On the other hand, an inhibitory effect of acupuncture-like stimulation on HR was attenuated by hypercapnia, which increases the tonic level of CSNA (Uchida et al., 2010). Therefore, HR response to somatosensory stimulation may differ, depending on the tonic CSNA level and resting HR.

### Types of somatosensory stimulation elicited by calf mechanical pressure

In the present study, mechanical stimulation was applied to the calf over the skin. Although, this would also activate cutaneous mechanoreceptors, HR responses were inducible even when the skin over the calf was removed. Also, mechanical skin stimulation (including noxious stimulation) applied around the calf induced only a small increase in HR (Kimura et al., 1995). In addition, the pressor response to calf mechanical pressure stimulation was diminished by severing the sciatic nerve (Stebbins et al., 1988), which is the main sensory transmission pathway from calf muscles. A possibility remains that afferents responding to blood vessel distortion elicited by the pressure stimulation may contribute to the cardiovascular responses (Cui et al., 2012).

Based on single unit recordings of group III and IV muscle afferents, the stimulation intensity used in the present study (10 N/cm^2^) could be considered either noxious (Berberich et al., 1988) or non-noxious (Hoheisel et al., 2005). Taguchi et al. (2005) stated that it was not possible to classify muscle afferents into low and high thresholds because the mechanical threshold of pressure stimulation is continuous. Hence, we are unable to define the stimulation used in the present study as noxious or non-noxious stimulation. Clinically, the intensity of touch-pressure stimulation for diagnosing myalgia of patients with temporomandibular joint disorders is 1 kg with a finger (Schiffman et al., 2014), estimated at 1 kg/cm^2^ (≈10N/cm^2^). Also, the pressure pain threshold on the head in healthy adults is ~4 kg/cm^2^ (Antonaci et al., 1998). Taken together, the stimulation intensity used in the present study may be non-noxious in animals without injuries. However, caution is necessary when extrapolating data obtained in humans to rats because pressure stimulation to deep tissues may be influenced by the thickness of subcutaneous tissues (Takahashi et al., 2005).

### Physiological significance

We suggest that these cardiovascular responses to mechanical pressure stimulation of skeletal muscles may help control intramuscular pressure through regulation of the blood supply. In this aspect, the role of pressure sensitive mechanoreceptors may differ from those of contraction- and stretch-sensitive mechanoreceptors that increase HR and blood pressure during exercise because an adequate stimulus for each type of mechanoreceptors is different. For example, compartment syndrome is a condition that causes muscle ischemic necrosis resulting from an excessive increase in intramuscular pressure. Because 100 mmHg is approximately equivalent to 1.3 N/cm^2^, force loading on muscle tissues is much greater under mechanical pressure stimulation used in the present study. Thus, such a cardiovascular response may protect muscles from potential damage by limiting blood perfusion partly due to regulating cardiac output, thereby preventing muscle edema and subsequently an abnormal intramuscular pressure. This potential function warrants further study.

In summary, the present results suggest that pressure stimulation applied to the calf excites muscle mechanoreceptors, that activate or inhibit CSNA, resulting in changes to HR. The direction of HR responses (tachycardia or bradycardia) to calf stimulation is determined by the tonic level of CSNA, suggesting that this mechanical stimulation-induced cardiovascular reflex participates in bidirectional feedback regulation of muscle blood supply.

## Author contributions

NW contributed to study design, data acquisition, data analysis, data interpretation, and manuscript writing. HH contributed to study design, data acquisition, data interpretation, and manuscript writing. All authors approved the final version of the manuscript and agreed to be accountable for all aspects of the work in ensuring that questions related to the accuracy or integrity of any part of the work are appropriately investigated and resolved.

## Funding

The present study was supported by JSPS KAKENHI (grant number JP25871216).

### Conflict of interest statement

The authors declare that the research was conducted in the absence of any commercial or financial relationships that could be construed as a potential conflict of interest.

## Abbreviations

CSNA: cardiac sympathetic efferent nerve activity
CVLM: caudal ventrolateral medulla
ΔCSNA: cardiac sympathetic efferent nerve activity response, ΔHR, heart rate response
EEG: electroencephalogram
HR: heart rate
NTS: nucleus of the solitary tract
RVLM: rostral ventrolateral medulla.

## References

[B1] Albrecht M. Henke J. Tacke S. Markert M. Guth B. (2014). Influence of repeated anaesthesia on physiological parameters in male Wistar rats: a telemetric study about isoflurane, ketamine-xylazine and a combination of medetomidine, midazolam and fentanyl. BMC Vet. Res. 10:310. 10.1186/s12917-014-0310-8 25551200 PMC4301083

[B2] Antonaci F. Sand T. Lucas G. A. (1998). Pressure algometry in healthy subjects: inter-examiner variability. Scand. J. Rehabil. Med. 30, 3–8. 9526748 10.1080/003655098444255

[B3] Araki T. Ito K. Kurosawa M. Sato A. (1984). Responses of adrenal sympathetic nerve activity and catecholamine secretion to cutaneous stimulation in anesthetized rats. Neuroscience 12, 289–299. 6462449 10.1016/0306-4522(84)90154-4

[B4] Berberich P. Hoheisel U. Mense S. (1988). Effects of a carrageenan-induced myositis on the discharge properties of group III and IV muscle receptors in the cat. J. Neurophysiol. 59, 1395–1409. 3385466 10.1152/jn.1988.59.5.1395

[B5] Campos R. R. McAllen R. M. (1997). Cardiac sympathetic premotor neurons. Am. J. Physiol. 272(2 Pt 2), R615–R620. 9124486 10.1152/ajpregu.1997.272.2.R615

[B6] Coote J. H. Hilton S. M. Perez-Gonzalez J. F. (1971). The reflex nature of the pressor response to muscular exercise. J. Physiol. 215, 789–804. 5090995 10.1113/jphysiol.1971.sp009498 PMC1331914

[B7] Cui J. McQuillan P. M. Blaha C. Kunselman A. R. Sinoway L. I. (2012). Limb venous distension evokes sympathetic activation via stimulation of the limb afferents in humans. Am. J. Physiol. Heart Circ. Physiol. 303, H457–H463. 10.1152/ajpheart.00236.2012 22707559 PMC3423143

[B8] Dampney R. A. L. Horiuchi J. Tagawa T. Fontes M. A. P. Potts P. D. Polson J. W. (2003). Medullary and supramedullary mechanisms regulating sympathetic vasomotor tone. Acta Physiol. Scand. 177, 209–218. 10.1046/j.1365-201X.2003.01070.x 12608991

[B9] Degtyarenko A. M. Kaufman M. P. (2006). Barosensory cells in the nucleus tractus solitarius receive convergent input from group III muscle afferents and central command. Neuroscience 140, 1041–1050. 10.1016/j.neuroscience.2006.02.050 16626870

[B10] Delaunois A. Dedoncker P. Hanon E. Guyaux M. (2009). Repeated assessment of cardiovascular and respiratory functions using combined telemetry and whole-body plethysmography in the rat. J. Pharmacol. Toxicol. Methods 60, 117–129. 10.1016/j.vascn.2009.07.003 19616108

[B11] Gibbs N. M. Larach D. R. Skeehan T. M. Schuler H. G. (1989). Halothane induces depressor responses to noxious stimuli in the rat. Anesthesiology 70, 503–510. 2923298 10.1097/00000542-198903000-00022

[B12] Gladwell V. F. Fletcher J. Patel N. Elvidge L. J. Lloyd D. Chowdhary S.. (2005). The influence of small fibre muscle mechanoreceptors on the cardiac vagus in humans. J. Physiol. 567(Pt 2), 713–721. 10.1113/jphysiol.2005.089243 15946971 PMC1474211

[B13] Graven-Nielsen T. Mense S. Arendt-Nielsen L. (2004). Painful and non-painful pressure sensations from human skeletal muscle. Exp. Brain Res. 159, 273–283. 10.1007/s00221-004-1937-7 15480607

[B14] Guyenet P. G. (2006). The sympathetic control of blood pressure. Nat. Rev. Neurosci. 7, 335–346. 10.1038/nrn1902 16760914

[B15] Hoheisel U. Unger T. Mense S. (2005). Excitatory and modulatory effects of inflammatory cytokines and neurotrophins on mechanosensitive group IV muscle afferents in the rat. Pain 114, 168–176. 10.1016/j.pain.2004.12.020 15733642

[B16] Horiuchi J. Killinger S. Dampney R. A. L. (2004). Contribution to sympathetic vasomotor tone of tonic glutamatergic inputs to neurons in the RVLM. Am. J. Physiol. Regul. Integr. Comp. Physiol. 287, R1335–R1343. 10.1152/ajpregu.00255.2004 15271655

[B17] Hotta H. Schmidt R. F. Uchida S. Watanabe N. (2010a). Gentle mechanical skin stimulation inhibits the somatocardiac sympathetic C-reflex elicited by excitation of unmyelinated C-afferent fibers. Eur. J. Pain 14, 806–813. 10.1016/j.ejpain.2010.02.009 20303306

[B18] Hotta H. Watanabe N. Orman R. Stewart M. (2010b). Efferent and afferent vagal actions on cortical blood flow and kainic acid-induced seizure activity in urethane anesthetized rats. Auton. Neurosci. 156, 144–148. 10.1016/j.autneu.2010.04.010 20510656

[B19] Imai K. Kitakoji H. (2003). Comparison of transient heart rate reduction associated with acupuncture stimulation in supine and sitting subjects. Acupunct. Med. 21, 133–137. 10.1136/aim.21.4.133 14740810

[B20] Kannan H. Yamashita H. Koizumi K. Brooks C. M. (1988). Neuronal activity of the cat supraoptic nucleus is influenced by muscle small-diameter afferent (groups III and IV) receptors. Proc. Natl. Acad. Sci. U.S.A. 85, 5744–5748. 3399508 10.1073/pnas.85.15.5744 PMC281838

[B21] Kaufman A. Sato A. Sato Y. Sugimoto H. (1977). Reflex changes in heart rate after mechanical and thermal stimulation of the skin at various segmental levels in cats. Neuroscience 2, 103–109. 917269 10.1016/0306-4522(77)90071-9

[B22] Kimura A. Ohsawa H. Sato A. Sato Y. (1995). Somatocardiovascular reflexes in anesthetized rats with the central nervous system intact or acutely spinalized at the cervical level. Neurosci. Res. 22, 297–305. 7478293 10.1016/0168-0102(95)00907-b

[B23] Koba S. Xing J. Sinoway L. I. Li J. (2008). Sympathetic nerve responses to muscle contraction and stretch in ischemic heart failure. Am. J. Physiol. Heart Circ. Physiol. 294, H311–H321. 10.1152/ajpheart.00835.2007 17965282

[B24] Manabe N. Foldes F. F. Töröcsik A. Nagashima H. Goldiner P. L. Vizi E. S. (1991). Presynaptic interaction between vagal and sympathetic innervation in the heart: modulation of acetylcholine and noradrenaline release. J. Auton. Nerv. Syst. 32, 233–242. 1645381 10.1016/0165-1838(91)90117-l

[B25] Matsukawa K. Wall P. T. Wilson L. B. Mitchell J. H. (1990). Reflex responses of renal nerve activity during isometric muscle contraction in cats. Am. J. Physiol. 259(5 Pt 2), H1380–H1388. 2240239 10.1152/ajpheart.1990.259.5.H1380

[B26] Matsukawa K. Wall P. T. Wilson L. B. Mitchell J. H. (1994). Reflex stimulation of cardiac sympathetic nerve activity during static muscle contraction in cats. Am. J. Physiol. 267(2 Pt 2), H821–H827. 8067438 10.1152/ajpheart.1994.267.2.H821

[B27] McCord J. L. Kaufman M. P. (2010). Reflex autonomic responses evoked by group III and IV muscle afferents, in Translational Pain Research: from Mouse to Man, 1st Edn., eds Kruger L. Light A. R. (Boca Raton, FL: CRC Press), 283–299. 21882465

[B28] Mense S. Meyer H. (1985). Different types of slowly conducting afferent units in cat skeletal muscle and tendon. J. Physiol. 363, 403–417. 4020704 10.1113/jphysiol.1985.sp015718 PMC1192937

[B29] Mizumura K. Taguchi T. (2016). Delayed onset muscle soreness: involvement of neurotrophic factors. J. Physiol. Sci. 66, 43–52. 10.1007/s12576-015-0397-0 26467448 PMC10716961

[B30] Murphy M. N. Mizuno M. Mitchell J. H. Smith S. A. (2011). Cardiovascular regulation by skeletal muscle reflexes in health and disease. Am. J. Physiol. Heart Circ. Physiol. 301, H1191–H1204. 10.1152/ajpheart.00208.2011 21841019 PMC3197431

[B31] Ohsawa H. Okada K. Nishijo K. Sato Y. (1995). Neural mechanism of depressor responses of arterial pressure elicited by acupuncture-like stimulation to a hindlimb in anesthetized rats. J. Auton. Nerv. Syst. 51, 27–35. 7722213 10.1016/0165-1838(95)80004-t

[B32] Overton J. M. (1993). Influence of autonomic blockade on cardiovascular responses to exercise in rats. J. Appl. Physiol. 75, 155–161. 8376262 10.1152/jappl.1993.75.1.155

[B33] Paintal A. S. (1960). Functional analysis of group III afferent fibres of mammalian muscles. J. Physiol. 152, 250–270. 14429833 10.1113/jphysiol.1960.sp006486 PMC1363314

[B34] Ruggeri P. Ermirio R. Molinari C. Calaresu F. R. (1995). Role of ventrolateral medulla in reflex cardiovascular responses to activation of skin and muscle nerves. Am. J. Physiol. 268(6 Pt 2), R1464–R1471. 7611523 10.1152/ajpregu.1995.268.6.R1464

[B35] Sato A. Sato Y. Schmidt R. F. (1981). Heart rate changes reflecting modifications of efferent cardiac sympathetic outflow by cutaneous and muscle afferent volleys. J. Auton. Nerv. Syst. 4, 231–247. 7299040 10.1016/0165-1838(81)90047-3

[B36] Sato A. Sato Y. Schmidt R. F. (1982). Changes in heart rate and blood pressure upon injection of algesic agents into skeletal muscle. Pflügers Arch. 393, 31–36. 7088681 10.1007/BF00582387

[B37] Sato A. Sato Y. Schmidt R. F. (1997). The impact of somatosensory input on autonomic functions. Rev. Physiol. Biochem. Pharmacol. 130, 1–328. 9019119

[B38] Schiffman E. Ohrbach R. Truelove E. Look J. Anderson G. Goulet J. P.. (2014). Diagnostic criteria for temporomandibular disorders (DC/TMD) for clinical and research applications: recommendations of the international RDC/TMD consortium network and orofacial pain special interest group. J. Oral Facial Pain Headache 28, 6–27. 10.11607/jop.1151 24482784 PMC4478082

[B39] Sharp J. Azar T. Lawson D. (2014). Effects of a complex housing environment on heart rate and blood pressure of rats at rest and after stressful challenges. J. Am. Assoc. Lab. Anim. Sci. 53, 52–60. 24411780 PMC3894648

[B40] Stebbins C. L. Brown B. Levin D. Longhurst J. C. (1988). Reflex effect of skeletal muscle mechanoreceptor stimulation on the cardiovascular system. J. Appl. Physiol. 65, 1539–1547. 3182517 10.1152/jappl.1988.65.4.1539

[B41] Suzuki A. Uchida S. Hotta H. (2004). The effects of aging on somatocardiac reflexes in anesthetized rats. Jpn. J. Physiol. 54, 137–141. 10.2170/jjphysiol.54.137 15182420

[B42] Taguchi T. Sato J. Mizumura K. (2005). Augmented mechanical response of muscle thin-fiber sensory receptors recorded from rat muscle-nerve preparations *in vitro* after eccentric contraction. J. Neurophysiol. 94, 2822–2831. 10.1152/jn.00470.2005 16160095

[B43] Takahashi K. Taguchi T. Itoh K. Okada K. Kawakita K. Mizumura K. (2005). Influence of surface anesthesia on the pressure pain threshold measured with different-sized probes. Somatosens. Mot. Res. 22, 299–305. 10.1080/08990220500420475 16503582

[B44] Tallarida G. Baldoni F. Peruzzi G. Raimondi G. Massaro M. Sangiorgi M. (1981). Cardiovascular and respiratory reflexes from muscles during dynamic and static exercise. J. Appl. Physiol. Respir. Environ. Exerc. Physiol. 50, 784–791. 7263361 10.1152/jappl.1981.50.4.784

[B45] Terui N. Saeki Y. Kumada M. (1987). Confluence of barosensory and nonbarosensory inputs at neurons in the ventrolateral medulla in rabbits. Can. J. Physiol. Pharmacol. 65, 1584–1590. 3690391 10.1139/y87-249

[B46] Uchida S. Hotta H. Kagitani F. Aikawa Y. (2003). Ovarian blood flow is reflexively regulated by mechanical afferent stimulation of a hindlimb in nonpregnant anesthetized rats. Auton. Neurosci. 106, 91–97. 10.1016/s1566-0702(03)00073-0 12878077

[B47] Uchida S. Kagitani F. Watanabe N. Hotta H. (2010). Sympatho-inhibitory response of the heart as a result of short-term acupuncture-like stimulation of the rat hindlimb is not augmented when sympathetic tone is high as a result of hypercapnia. J. Physiol. Sci. 60, 221–225. 10.1007/s12576-009-0084-0 20087705 PMC10717387

[B48] Vanhoutte P. M. Levy M. N. (1980). Prejunctional cholinergic modulation of adrenergic neurotransmission in the cardiovascular system. Am. J. Physiol. 238, H275–H281. 6245589 10.1152/ajpheart.1980.238.3.H275

[B49] Vissing J. Wilson L. B. Mitchell J. H. Victor R. G. (1991). Static muscle contraction reflexly increases adrenal sympathetic nerve activity in rats. Am. J. Physiol. 261(5 Pt 2), R1307–R1312. 1951780 10.1152/ajpregu.1991.261.5.R1307

[B50] Watanabe N. Piché M. Hotta H. (2015). Types of skin afferent fibers and spinal opioid receptors that contribute to touch-induced inhibition of heart rate changes evoked by noxious cutaneous heat stimulation. Mol. Pain 11, 4. 10.1186/s12990-015-0001-x 25884917 PMC4335417

[B51] Yli-Hankala A. Jäntti V. (1990). EEG burst-suppression pattern correlates with the instantaneous heart rate under isoflurane anaesthesia. Acta Anaesthesiol. Scand. 34, 665–668. 2275329 10.1111/j.1399-6576.1990.tb03169.x

