# Supplementary material for: Heart Rate Changes in Response to Mechanical Pressure Stimulation of Skeletal Muscles Are Mediated by Cardiac Sympathetic Nerve Activity
Source: Front Neurosci. 2017 Jan 10;10:614. doi: 10.3389/fnins.2016.00614 (PMC5222799; doi:10.3389/fnins.2016.00614)
Supplement: Supplementary file 1 [file DataSheet1.DOCX]

Supplementary Material

Heart Rate Changes in Response to Mechanical Pressure Stimulation of Skeletal Muscles are Mediated by Cardiac Sympathetic Nerve Activity

Nobuhiro Watanabe^1^, Harumi Hotta^1*^

*** Correspondence:** Harumi Hotta: hhotta@tmig.or.jp

# Supplementary Figure

## Supplementary Figures


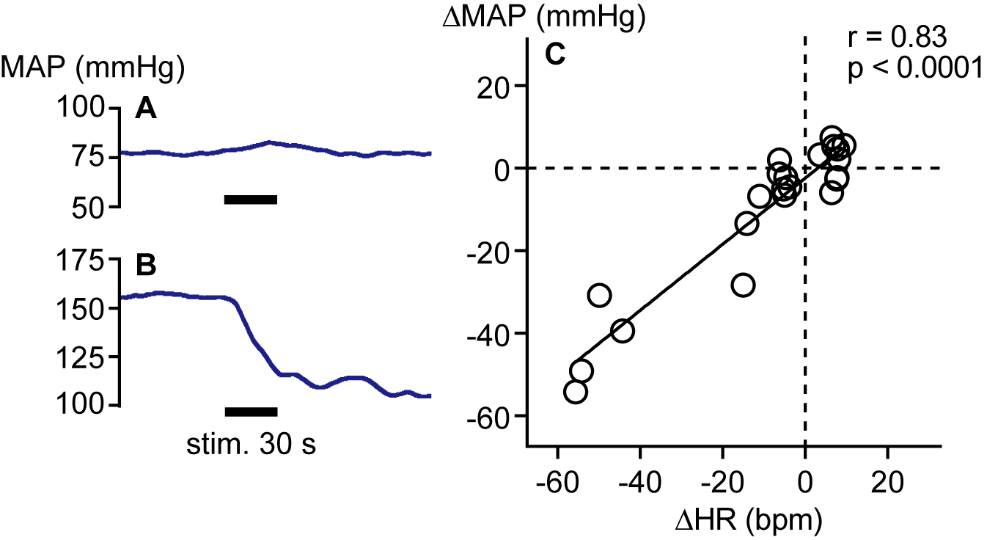


**Supplementary Figure 1.** Mean arterial pressure responses (ΔMAP) to calf pressure stimulation in parallel with heart rate responses (ΔHR). (**A, B**) Sample records increased (**A**) or decreased (**B**) MAP. The presented records (**A** and **B**) were obtained in the same trials shown in Figure 1**A** and 1**B**, respectively. (**C**) Positive correlation between ΔHR and ΔMAP. r = Spearman’s correlation coefficient.
